# Coexistence of Hashimoto's thyroiditis and papillary thyroid carcinoma revisited in thyroidology, an experience from an endemic region: fad or future?

**DOI:** 10.1590/1806-9282.20231380

**Published:** 2024-05-13

**Authors:** Ilkay Cinar, Ilker Sengul

**Affiliations:** 1 Giresun University, Faculty of Medicine Department of Pathology – Giresun, Turkey.; 2 Giresun University, Faculty of Medicine, Division of Endocrine Surgery – Giresun, Turkey.; 3 Giresun University, Faculty of Medicine, Department of General Surgery – Giresun, Turkey.

**Keywords:** Thyroid gland, Thyroid cancer, papillary, Thyroiditis, pathology, Thyroidology

## Abstract

**OBJECTIVE:**

Papillary thyroid carcinoma, per se, is the most common type of thyroid cancer, and Hashimoto's thyroiditis is the most frequent autoimmune disease of the papillon gland. The liaison between Hashimoto's thyroiditis and thyroid cancers is still an ongoing debate in thyroidology. The aim of the study was to discuss the frequency of the co-occurrence of Hashimoto's thyroiditis and papillary thyroid carcinoma.

**METHODS:**

This study is designed as a retrospective analytical cohort study. The institutional database and archive of histopathology scanning identified the patients who had undergone thyroidectomy between January 2022 and January 2016. The Statistical Package for Social Sciences v21.0 program was used for statistical purposes. Descriptive and chi-square tests were applied, and a p<0.05 was considered significant.

**RESULTS:**

Of 498 patients who had undergone thyroidectomy for 4 years, 99 (20%) were male and 399 (80%) were female. Of note, papillary thyroid carcinoma was revealed in 160 (32%) patients, and Hashimoto's thyroiditis was recognized in 178 (35.74%) patients. The prevalence of Hashimoto's thyroiditis in cases with papillary thyroid carcinoma was 43.8%, while the prevalence in patients with Hashimoto's thyroiditis was 41.1%.

**CONCLUSION:**

A debate still remains on the propriety of these two phenomena. Herewith, we recognized a correlation between the presence of papillary thyroid carcinoma and Hashimoto's thyroiditis. Providers should be vigilant about the coexistence of these phenomena. We might postulate the so-called total thyroidectomy for cases with a cytologic diagnosis of Hashimoto's thyroiditis with a papillary thyroid carcinoma. As a matter of fact, this issue merits further investigation.

## INTRODUCTION

Thyroid carcinomas are the most common endocrine tumors, which occupy the ninth most frequent cancer worldwide, according to World Health Organization's 2020 data. However, it is more commonly found in Asian populations and is the fifth most common tumor in Turkey. Papillary thyroid carcinoma (PTC) is the most common thyroid cancer with a commonly good prognosis, although sometimes poor clinical outcomes emerge. As such, tall cell, cribriform, diffuse sclerosing, and hobnail subtypes are considered to have a poor prognosis for PTC^1^.

Hashimoto's thyroiditis (HThy), chronic lymphocytic thyroiditis, was first described by a Japanese pathologist and surgeon, Hakaru Hashimoto, in 1912^2^. It is the most common autoimmune thyroid disease, especially in females between the 3rd and 5th decades, possessing a prevalence of HThy of 1–4% and an incidence of 3–6 per 10,000 people. Lymphocyte infiltration and atrophy due to an autoimmune response in the gland are recognized in HThy, which is one of the most common causes of hypothyroidism that develops in 4–5% of cases each year^3^. The associations between PTC and HThy have been reported between the two phenomena^3,4-7^, though some publications^8-10^ are opposing.

The present study sought to determine the prevalence of thyroid carcinoma and HThy in the cases who had undergone thyroidectomy in the iodine-deficiency endemic region.

## METHODS

### Study design

This is a retrospective cohort study conducted at the Department of Pathology, Giresun University Education and Research Hospital, incorporating 498 patients with thyroidectomies between January 2016 and January 2022. The patient records have been obtained from the institutional database, and archive of histopathology for the purpose of collecting demographic data and histopathologic diagnoses. The tumor size, vascular invasion, multifocality, extrathyroidal extension, nodal metastasis, and subgroup of PTC have been evaluated. To this end, the patients were categorized into two groups: those with HThy and those with PTC. Afterward, the incidence of PTC was determined among the cases with HThy, and subsequently, the occurrence of HThy in patients with PTC was analyzed. Of note, the multifocality, lymphovascular invasion, and tumor diameter were assessed in cases with both HThy and PTC.

### Statistical analysis

All the patients’ data were entered into a Microsoft Excel (Microsoft Corporation, Redmond, WA, USA) spreadsheet and statistically analyzed using the Statistical Package for Social Sciences (SPSS) version 21.0 (SPSS, IBM Inc., Chicago, IL, USA) software in order to evaluate the present retrospective analytical cohort study. The data were statistically evaluated using the chi-square test and Fisher's exact test. A p<0.05 was considered statistically significant.

## RESULTS

In total, 99 (20%) of cases were male and 399 (80%) were female in 498 patients who had undergone thyroidectomy for six decades. The mean age was 51 (21–81) years, and the PTC was revealed in 160 (32%) and HT in 178 (35.74%) cases. However, 343 (68%) cases had thyroid follicular nodular diseases, while 14 (2.8%) had diffuse hyperplasia, 48 (9.6%) had follicular adenoma, 28 (5.5%) had Hurtle cell adenoma, 4 (0.8%) had follicular carcinoma, 4 (0.8%) had Hurtle cell carcinoma, and 13 (2.6%) had non-invasive follicular thyroid neoplasm with papillary-like nuclear features.

**Figure 1:**
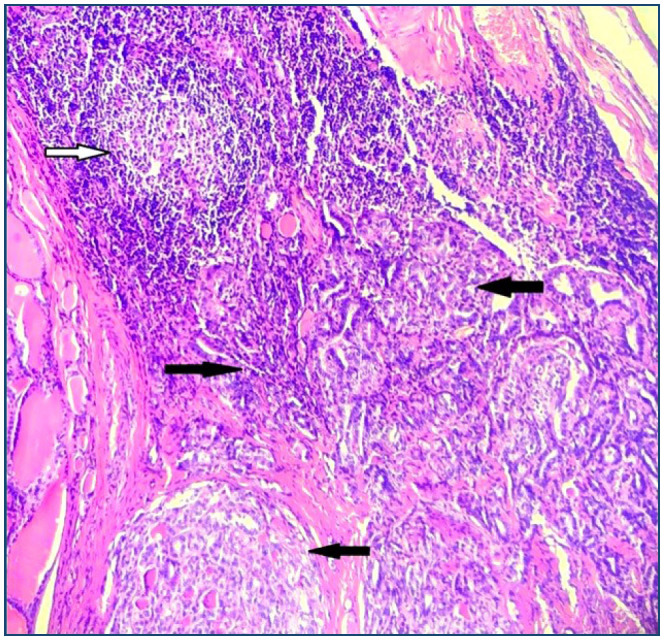
Papillary thyroid carcinoma in the background of Hashimoto's thyroiditis (the white arrow indicates a lymphoid follicle with a germinal center in Hashimoto's thyroiditis, while the black ones indicate papillary microcarcinoma near the lymphoid infiltration).

Histopathologic diagnosis was HThy and/or PTC in 268 of a total of 468 patients included in the study; 42 (15.7%) of them were male and 226 (84.3%) were female. Herein, tumor size was smaller than 10 mm in 104 (45.5%) and 10 mm or larger in 56 (20.9%) cases with PTC, in which the tumor was found multifocal in 44. Both PTC and HThy were present in 70 cases (Figure 1), and the prevalence of HThy in patients with PTC was 43.7% and that of PTC in HThy was revealed to be 39.3%. In this case, tumor size was smaller than 10 mm in 48 (68.6%) and multifocal in 22 (31.4%) (Table 1). Lymphovascular invasion was detected in six cases with PTC, and HThy was exhibited in half of them without significance (p=0.52). However, statistical significance was recognized in the case of coexistence of HThy with PTC cases, regardless of tumor size (p<0.001) (Table 2), in which tumors were multifocal in 22 of 70 cases. A statistical correlation was observed between multifocality and HThy (p=0.019). In addition, the odds ratio was calculated to be 2.3 in the coexistence of PTC and HThy.

**Table 1:** Demographic, sonographic, and histopathologic features of papillary thyroid carcinoma and Hashimoto's thyroiditis.

		PTC (n=160)	HThy (n=178)
Male		31 (19.4%)	23 (12.9%)
Female		129 (80.6%)	155 (87.1%)
Median age		51 (21–84)	51 (21–81)
HThy with PTC		70 (43.7%)	70 (39.3%)
Multifocality		44 (27.5%)	22 (31.4%)
Tumor size (cm)			
	≥1	58 (36.3%)	22 (31.4%)
	<1	102 (63.7%)	48 (68.6%)
Lymphovascular invasion		6 (8.6%)	3 (4.3%)
Lymph node metastasis		6 (8.6%)	3 (4.3%)

**Table 2:** Statistical outcomes of the cases with papillary thyroid carcinoma and/or Hashimoto's thyroiditis (n=268).

			HThy		Total
			Absent	Present	
PTC	Absent	Count	0	108	110
		% within PTC	0.0%	100.0%	100.0%
		% within HThy	0.0%	60.7%	40.3%
	Present	Count	90	70*	160
		% within PTC	56.2%	43.8%	100.0%
		% within HThy	100.0%	39.3%	59.7%
Total		Count	90	178	268
		% within PTC	33.6%	66.4%	100.0%
		% within HThy	100.0%	100.0%	100.0%
Odds ratio		Values	95% Confidence interval		
			Lower	Upper	
For cohort HThy present		2.324	1.942	2.780	
No. of valid cases		268			

* p<0.001 (Fisher's exact test).

## DISCUSSION

In this study, it was concluded that there is a correlation between HThy and PTC in the patient group examined. The size of tumors was mostly less than 10 mm in cases with HThy, and a tendency toward multifocal disease was observed. The presence of multiple small tumor islands in the background of HThy suggests that it might provoke the development of PTC. To date, various hypotheses have been put forward in studies on the subject. The Cappelli et al.'s^11^ study has linked high thyroid stimulating hormone (TSH) levels with an increased risk of malignancy. Based on this research, it may be thought that increased TSH secondary to hypothyroidism in thyroiditis might lead to increased follicular epithelial cell proliferation and thyroid papillary carcinoma. On the contrary, some studies argue that thyroid autoimmunity might emerge against antigens released by cancerous thyrocytes^7^. Most recent studies have reported correlations between the two diseases, whose outcomes support the results of this study. Of these, Liang et al., reported a significantly higher risk of PTC development in patients with HThy^12^. Uhliarova et al.^13^ stated that HThy causes a significantly increased risk of developing thyroid carcinoma, especially papillary thyroid microcarcinoma. Moreover, Molnár et al.^14^ reported that HThy is a promoter of thyroid carcinogenesis. The authors also described a correlation with tumoral multifocality^14^. In addition, a high rate of multicentricity in tumors in HThy has been reported in some studies in our country^15-19^. Although HThy is a possible risk factor for PTC, it has been suggested that it reduces progression^20^, especially in thyroidology^21-25^, a vital and substantial field in order to provide optimal thyroid health by thyroidologists; however, no such results were found in our study. Poor prognostic factors such as lymph node metastasis and lymphovascular invasion in the cases were not statistically different when compared to other cases. There have been reported associations between the studies and thyroidectomy specimens, but in some studies that were performed with fine needle aspiration cytology (FNAC) no correlation was reported between the two phenomena^8,9^.

## CONCLUSION

There has been an increase in the prevalence of PTC in HThy, although a debate is still ongoing about the provocation of these two phenomena. A non-negligible frequency of HThy and PTC coexistence and a noteworthy risk of multifocal disease should be considered in the management and follow-up of the phenomenon. As such, HThy should be followed up for the development of PTC, and we might postulate that the so-called surgical procedure of total thyroidectomy should be planned in patients with HThy with a PTC diagnosis in FNAC. As a matter of fact, this issue merits further investigation.
